# Convergence of disciplines: a systematic review of multidisciplinary development approaches in artificial intelligence

**DOI:** 10.3389/fdgth.2025.1400338

**Published:** 2025-08-13

**Authors:** Giusi Antonia Toto, Luca Grilli, Luigi Traetta, Rosanna Villani, Annamaria Petito, Gaetano Serviddio

**Affiliations:** Department of Human Science and Education, University of Foggia, Foggia, Italy

**Keywords:** artificial intelligence, multidisciplinary approach, medicine, psychology, agriculture, mathematics

## Abstract

The integration of artificial intelligence (AI) across multiple disciplines is fostering a transformative shift in research and practice. This paper explores how multidisciplinary collaboration with AI is reshaping traditional methodologies and catalyzing innovation in diverse fields such as medicine, psychology, agriculture, mathematics, physics, and economics. A systematic review was conducted following the PRISMA 2020 guidelines. Relevant literature was identified through searches in PubMed, Scopus, and Google Scholar, covering publications from 2013 to August 2023. Inclusion criteria focused on English-language articles examining the intersection of AI and multidisciplinary applications. Additional studies were identified by screening reference lists of included articles. The analysis revealed that AI's multidisciplinary integration has significantly influenced practices across multiple domains. In medicine, AI supports diagnosis and treatment planning; in psychology, it enhances mental health interventions; and in agriculture, it contributes to addressing global food security challenges. The reviewed literature highlights how AI collaboration with fields such as physics, economics, and history is leading to innovative problem-solving strategies and paradigm shifts. The findings underscore the substantial potential of a multidisciplinary approach to AI. This convergence is not only accelerating technological advancement but also fostering more comprehensive and effective solutions to complex global issues. The results suggest that ongoing interdisciplinary collaboration will be critical in maximizing AI's societal impact and shaping its future development.

## 1 Background

Artificial Intelligence involves machines and computer systems emulating human intelligence procedures (1). AI finds applications in various domains such as natural language processing, speech recognition, machine vision, and expert systems. In straightforward terms, AI blends computer science (2) with well-curated datasets to address specific challenges. Additionally, it includes subdomains like machine learning and deep learning, often associated with AI (3). These areas consist of algorithms designed to construct expert systems capable of predictions or classifications using input data.

**Table 1:** Study descriptor table.

Authors	Year of publication	Methodology	Participants	Domains and applications	Collaborative disciplines	AI techniques	Results and findings	limitations
Ahmad and Afzal	2022	Mixed	N/A	Analysis and detection support	Mathematical modeling	Newly developed plithogenic distance and similarity measures in a fuzzy environment.	The algorithm of the method not only detects the infection in the suspects but also explains its severity.	N/A
Ahuja et al.	2022	Qualitative	N/A	Medical imaging, diagnostic support	Ophthalmo logy	Artificial neural networks (ANNs)	Ophthalmic AI systems are advantageous in that they decrease the time required to interpret image data, enable ophthalmologists to Gain a greater understanding of disease progression and assist with early- stage diagnosis, staging, and prognosis.	Limited screening scope of AI-based systems.
Ben and Hanana	2021	Qualitative	N/A	Crop yield, soil properties, and irrigation requirements.	Agriculture	Artificial neural networks(ANNs)	The unquestionable growing tendency of Al and ML algorithms is the adoption of these algorithms to improve food industries.	High cost of creation and maintenance
Bird et al.	2018	Qualitative	213 health volunteers	Psychologi cal interventio n	Psycholog y	AI chabot; manage your life online (MYLO)	Programs were associated with improvements in problem distress, anxiety and depression post- intervention, and again 2 weeks later.	Study design (RCT). Use of non- validated instruments.
Butow and Hoque	2020	Qualitative	N/A	Healthcare, communication support	Healthcare	Supervised and unsupervised learning algorithms.	Application of artificial intelligence to communication skills training has been attempted, to provide audit and feedback, and through the use of avatars.	An individual may feel hesitant to seek help from a human trainer due to scheduling constraints, or the possible stigma involved in seeking more practice time.
Cho et al.	2020	Quantitativ e	43	Psychologi cal interventio n	Psycholog y	A circadian rhythm- based algorithm based on data obtained with a wearable activity tracker.	Positive changes in health behaviours due to the alerts and in wearable device adherence rates were observed in the circadian rhythm for mood (CRM) group.	The case- control sample was not sufficiently matched. Allocation to the CRM feedback intervention was nonrandomi zed. The Sham app correspondi ng to the CRM app feedback system was not completely provided to the control group. Light sensor data could not be collected from iPhone users for technical reasons, so this could not be analyzed.
Davies et al.	2021	Qualitative	N/A	Discovering new conjectures and theorems	Advancing mathematics	Supervised and attributed learning techniques	A new connection between the algebraic and geometric structure of knots, and a candidate algorithm predicted by the combinatorial invariance conjecture for symmetric groups.	N/A
Diaz et al.	2021	Mixed	219	Daily task support	Medical physics	AI software in medical devices	AI was perceived as a positive resource to support Medical Physics Experts (MPEs) in their daily tasks.	N/A
Elleuch et al.	2021	Qualitative	N/A	Medical care	Mathematical modelling & healthcare	Artificial neural networks	Combining both models [artificial neural networks methods, and operations research (OR) through a fuzzy interval mathematical model] provides an effective assessment under scarce initial information to select a suitable list of patients for a set of hospitals.	N/A
Ferreira et al.	2020	Mixed	N/A	Breast cancer care	Medicine	Transfer learning using pre- trained models	Potential benefits for A in medicine of introducing future scientists, engineers, and clinicians to cross- cultural design- thinking early in their educational experiences.	N/A
Fulmer et al.	2028	Qualitative	74	CBT and other interventions	Psycholog y	AI chatbot: conversatio nal Tess app	AI serves as a cost- effective and accessible therapeutic agent.	N/A
Green et al.	2020	Mixed	41	CBT interventio n	Psycholog y	AI chatbot: conversatio nal Zuri app, Kenyan version of Tess	Most interviewees who tried Zuri reported having a positive attitude toward the service and expressed trust in Zuri. They also attributed positive life changes to the intervention.	Only offered screening and conversations in English.
Gruson et al.	2020	Qualitative	N/A	Cardiology, diagnostic and prognostic support	Cardiovascular medicine	Biomarkers discovery and better understanding of complex disease pathways. Data aggregation and integration of omics. Resources stewardship p and demand management nt. Real-time monitoring of diseases or risk component s. Engines for early risk estimation. Improving diagnosis of AF, ACS, AMI or HF. Enhanced precision care and outcomes monitoring.	AI is providing new tools to improve research but also to enhance early risk estimation of diseases and diagnostic performanc es.	N/A
Hu et al.	2022	Qualitative	N/A	Diagnosis and treatment support	Hematopat hology	Convolutio nal neural network (CNN), recurrent neural network (RNN), long short- term memory (LSTM)	AI greatly promotes and standardize s the diagnostic process to complement and assist human activities in this field.	The model used in AI requires a large number of parameters, such as network topology, initial values of weights and thresholds. The learning process cannot be observed, and the output results are difficult to explain, which will affect the reliability and acceptability of the results.
Inkster et al.	2018	Mixed	129	CBT together with other interventions via the Wysa app	Psycholog y	AI-chatbot: conversatio nal Wysa app	Group had significantly higher average improveme nt compared with the Low users group.	The study design and statistical limitations.
Liu et al.	2022	Quantitativ e	83	CBT interventio n	Psycholog y	AI chatbot: conversatio nal XioNan app)	The chatbot- delivered self-help depression intervention was proven to be superior to the minimal level of bibliotherapy in terms of reduction of depression, anxiety, and therapeutic alliance achieved with participants.	The content of the chatbot was limited. The study recruited a participant sample that lacked variety.
Luo et al.	2021	Quantitativ e	150	Detection and prevention of cancer.	Gastroente rology	CNN algorithm, specifically, a YOLO network architecture for object detection.	A real-time automatic polyp detection system can increase the polyp detection rate (PDR), primarily for diminutive polyps.	A small sample size.
Lutz et al.	2019	Quantitativ e	1,234	CBT via trier treatment navigator	Psycholog y	Random forest algorithm.	The prediction of optimal treatment strategies resulted in differential prediction models substantiall y improving effect sizes and reliable improveme nt rates.	N/A
Makino et al.	2019	Quantitativ e	N/A	Medical, predictive model for diabetic kidney diseases	Medicine	Convolutio nal auto encode r	AI could predict DKD aggravation with 71% accuracy. The new predictive model by AI could detect the progression of DKD and may contribute to more effective and accurate interventions to reduce hemodialys is.	Information obtained from each EMR, especially from the medical doctors’ records, varies considerably and we could not unify the data extraction from each patient. The duration between each laboratory test was not uniform and depended on the individual patient. The study was carried out in a single centre and has not been reevaluated using EMR from other institutions. The study could not find any relationship between the progression of DKD for 6 months and medication
Montazeri et al.	2023	Qualitative	N/A	Fate, transport, and estimation of chromium from its point of discharge into the river until it is absorbed by agricultural products.	Agriculture, machine learning	Linear regression (LR), neural network (NN) classifier, and NN regressor	NN regressor is the most accurate model, followed by the LR, for estimating Cr levels in tomato leaves.	N/A
Nishida and Kudo	2023	Qualitative	N/A	Medical imaging, diagnosis and management of liver diseases	Ultrasonog raphy	Convolutional neural network	A variety of AI models for the diagnosis of liver disease have been reported; some of them reportedly outperform human experts.	N/A
Piette et al.	2022	Quantitativ e	278	Cognitive behavioural therapy intervention	Interactive voice response, therapy	Reinforce ment learning algorithm LinUCB	A comparativ e effectivene ss trial of AI-CBT-CP found that its outcomes were not inferior to those of 45-minute telephone therapist sessions, with less than half the therapist time.	The results of both interventions were relatively modest. Patients were recruited from the US VA health care system, and how the trial implementation and findings would be different if conducted in other settings is difficult to anticipate.
Reddy et al.	2019	Qualitative	N/A	Patient administration, clinical decision support, patient monitoring and healthcare interventions	Healthcare	Deep neural networks, natural language processing, computer vision and robotics	AI will be used extensively in healthcare delivery and there is huge potential for cost- saving as well as service quality improvement -.	N/A
Rondonotti et al.	2023	Quantitativ e	389	Optical diagnosis	Gastroente rology	Real-time AI system (CAD- EYE)	AI-assisted optical diagnosis matches the required PIVI thresholds. This does not however offset the need for endoscopis ts’ high level of confidence and expertise. The AI system seems to be useful, especially for no experts.	Inherent psychologic al bias, the multicenter design of the study and the performance of optical diagnosis without magnification.
Sadeh- Sharvit et al.	2023	Quantitativ e	47	Behavioral health care services support	Psycholog y	Health insurance portability and accountabi lity act– compliant, secure, password- protected Eleos health platform.	Mental health services provided in community -based clinics with an AI platform specializing in behavioura l treatment were more effective in reducing key symptoms than standard therapy.	The therapy sessions provided by the therapists in the TAU group were not captured by the AI platform, and therefore, it is impossible to assess whether these therapists used other decision- support tools to improve practice. Small sample size and the length of study.
Sezgin	2023	Qualitative	N/A	Diagnostic accuracy, optimized treatment planning, and improved patient outcomes.	Healthcare, large language models (LLMs)	Generative AI models	AI can create a paradigm shift in healthcare by complementing and enhancing the skills of healthcare providers, ultimately leading to improved service quality, patient outcomes, and a more efficient healthcare system.	N/A
Shandhi and Dunn	2022	Qualitative	N/A	Personalize d care, early disease detection, clinical decision support and tracking of disease progression	Personalize d and precision medicine, machine learning	Reinforcement learning algorithms, artificial neural networks, recurrent neural networks, convolutional neural networks and variational autoencode rs	The synergy between AI and personalize d/precision medicine could ultimately decrease the disease burden for the public at large, and, therefore, the cost of preventable health care for all.	Fairness and bias. Limited data availability. Data deluge. Transparenc y and liability. Data drift. Data safety and privacy. Trust.
Sohrabpou r et al.	2020	Qualitative	N/A	Sales forecasting	Economics	Genetic programming model	A high accuracy and predictive precision model.	A limited source of Data was obtained
Sonoda et al.	2021	Quantitativ e	413	Medical imaging	Radiology	Vector machine (SVM) model, Xception model and random forest model	AI algorithm can automatically classify with ambiguous criteria the presence or absence of a symmetric al blood vessel running pattern of the choroid. The classification was as good as that of supervised humans in accuracy and reproducibi lity.	The study population consisted of healthy eyes and diseased eyes and Each had different backgrounds such as age, refractive power, and axial length. The study was performed at a single institution with only two evaluators.
Wahle et al.	2016	Quantitativ e	126	CBT intervention + mobile sensing and support (MOSS) app	Psycholog y	LASSO (least absolute shrinkage and selection operator)	Binary classificati on performanc e for biweekly PHQ-9 samples (*n* = 143, with a cutoff of PHQ-9 ≥ 11, based on random forest and support vector machine leave-one- out cross- validation resulted in 60.1% and 59.1% accuracy, respectivel y.	The clinical study carried out is based on a nonrandomi zed, uncontrolle d single-arm study design, which rules out the possibility of proving a direct causal link between symptom improvement and MOSS app use. Subjects were not asked to provide information about relevant control variables. Not all subjects with an elevated PHQ-9 are certain to have depression. Did not quantify the efficacy of the proposed recommend ation algorithm.
Williams et al.	2018	Qualitative	N/A	Image diagnostic support	Physiologi cal genomics and precision medicine	Watson, deep neural networks.	Physiological genomic readouts in disease- relevant tissues, combined with advanced AI, can be a powerful approach for precision medicine for common diseases.	N/A

AF, Atrial fibrillation; ACS, acute coronary syndrome; AMI, acute myocardial infarction; HF, heart failure; AI-CBT-CP, cognitive behavioural therapy intervention for chronic pain; CBT, cognitive behavioural therapy; CAD, computer-aided diagnosis; EMR, electronic medical records.

The landscape of artificial intelligence has evolved remarkably over the past few decades. In the previous instance when generative AI held such prominence, significant advancements occurred within computer vision (4). However, the current leap forward centres around natural language processing. Furthermore, this progress isn't confined to language alone; generative models now can grasp the structure of software code, molecules, real-world images, and various other data forms (5). From its early origins as a computational discipline rooted in computer science, AI has undergone a profound transformation, expanding beyond the confines of its birthplace to embrace a truly multidisciplinary approach. This shift is marked by a convergence of expertise from diverse fields, resulting in a collaborative effort that transcends traditional boundaries. The multidisciplinary approach to AI development holds immense significance in the contemporary technological landscape (6). As AI systems become increasingly sophisticated and integrated into various facets of human life, understanding their capabilities, limitations, and ethical implications is paramount.

The multidisciplinary approach to AI development is of paramount importance in the ever-evolving technological landscape. By integrating expertise from a wide array of disciplines, this approach not only deepens the understanding of AI but also significantly accelerates innovation. The infusion of diverse perspectives enriches the creative process, leading to breakthroughs that might not be possible within the constraints of a single discipline. Moreover, this collaborative synergy is crucial for tackling the intricate challenges posed by AI, which often require insights from multiple fields to be fully understood and effectively addressed.

The importance of investigating the multidisciplinary approach to AI cannot be overstated. Its transformative impact on research, innovation, and application extends far beyond traditional boundaries. As AI increasingly integrates into sectors as varied as healthcare, mathematics, art, and agriculture, the interdisciplinary approach amplifies its capacity to address complex, real-world problems. This broadened perspective enables AI to transcend its traditional applications, unlocking new potentials and driving forward the frontier of what technology can achieve in solving the most pressing global challenges.

In medical practice, artificial intelligence (AI) is becoming increasingly important. The challenge for physicians is to try to maintain an overview of the potential applications and usefulness of AI so that it can be applied efficiently and safely in healthcare. In agriculture, AI is applied through machine learning algorithms that can improve and support farmers’ agronomic practices, with the use of new machinery or support for existing machinery. In the cultural and artistic context, AI brings innovations in both the creation of artistic works and the evaluation of artistic structures and paths.

While numerous studies have explored AI from various angles, a comprehensive review focused on the multidisciplinary approach is conspicuously absent. Existing literature often delves into the technical aspects of AI, neglecting the intricate interplay between disciplines that catalyzes its evolution. This systematic review sought to accomplish the primary objective of examining and analyzing the emergence of the multidisciplinary approach to AI development. The aim was to uncover the layers of collaboration and integration that have driven AI into a domain far surpassing its computational beginnings. Through an exploration of how diverse disciplines contribute to the advancement of AI technologies, this review aimed to encapsulate the essence of this convergence and illuminate the benefits it brings to the forefront.

### 1.1 Research question

This paper aims to fill the aforementioned gap by performing a systematic review guided by the research question: How has the adoption of a multidisciplinary approach influenced the development of artificial intelligence in diverse fields, including medicine, psychology, history, physics, and other disciplines, and what notable outcomes have been achieved in recent years?

## 2 Methods

This review adhered to the PRISMA (Preferred Reporting Items for Systematic Reviews and Meta-Analyses) 2020 recommendations (7). A comprehensive search was conducted across two major databases, PubMed and Scopus, focusing on English-language articles published between 2013 and August 2023. The review specifically targeted studies that explore the intersection of artificial intelligence and its application across multidisciplinary fields, aiming to address a gap in the existing literature by providing insights into how a multidisciplinary approach influences the development and implementation of AI in various domains.

### 2.1 Primary search

A comprehensive strategy was employed across PubMed and Scopus databases. This strategy involved employing a blend of freely chosen keywords to maximize the sensitivity of the search. Specifically, a meticulous analysis of the complete text was conducted to ensure a thorough search approach. In PubMed and Scopus, the search strategies encompassed the following queries: (“Artificial intelligence” OR AI) AND (“mathematics” OR “mathematical” OR “quantitative” OR “modeling” OR “data analysis”) AND (“physics” OR “physical” OR “quantum” OR “theoretical physics”) (“Artificial intelligence” OR AI) AND (“medicine” OR “medical” OR “healthcare” OR “clinical” OR “biomedical”) AND (“psychology” OR “psychological” OR “behavioral” OR “cognitive” OR “emotional”) (“Artificial intelligence” OR AI) AND (“history” OR “historical” OR “historiography” OR “historical analysis”) AND (“social sciences” OR “sociology” OR “societal” OR “social behavior”).

### 2.2 Eligibility criteria

All studies had to meet the following predefined inclusion criteria:

• Original studies;
• Studies published from 2013 to August 2023, reflecting the most recent research up to the present;
• Focus on Mustidisciplinary AI;
• Studies that contribute to the understanding of how multidisciplinary collaboration shapes AI development;
• Published in English.
• Studies that satisfied the following criteria were excluded:
• Studies not directly related to the multidisciplinary approach in AI development, including studies that solely focus on technical aspects of AI without interdisciplinary collaboration;
• Non-English studies;
• Non-peer-reviewed sources, Reviews, editorials, conference papers, comments;
• Abstracts or summaries without sufficient detail to evaluate relevance to the review objectives;
• Studies solely concentrating on a single aspect of AI (e.g., natural language processing, computer vision) without discussing its multidisciplinary integration.

### 2.3 Study selection and data extraction

Potentially qualified studies underwent individual assessment using Zotero software. The process entailed meticulous evaluation of titles, abstracts, and complete texts. The comprehensive analysis of full texts centred on the presentation of any type of information concerning the multidisciplinary approach to artificial intelligence. Following the identification of articles for incorporation, pertinent data was extracted into a predefined data descriptor table containing the subsequent categories: author, publication year, research methodology, participants, application domain, collaborative disciplines, AI techniques, results/findings, and limitations.

### 2.4 Protocol registration

To ensure transparency and methodological rigor, this systematic review protocol was registered in PROSPERO. Registration provides a public record of the research objectives, eligibility criteria, and methodology established before commencing the review, reducing the risk of bias and enhancing reproducibility. Following established guidelines, this registration outlines the scope, search strategy, inclusion and exclusion criteria, and planned analyses to provide a clear framework for the review process.

### 2.5 Bias assessment

To evaluate the reliability and quality of the included studies, a formal bias assessment was conducted using validated tools. For randomized controlled trials, the Risk of Bias 2 (RoB 2) tool was applied, assessing domains such as randomization, deviations from intended interventions, missing outcome data, measurement of the outcome, and selection of the reported result. For non-randomized studies, the Risk Of Bias In Non-randomized Studies of Interventions (ROBINS-I) tool was utilized, examining biases arising from confounding, selection of participants, measurement of interventions, missing data, and selective reporting.

## 3 Results

**Figure 1:**
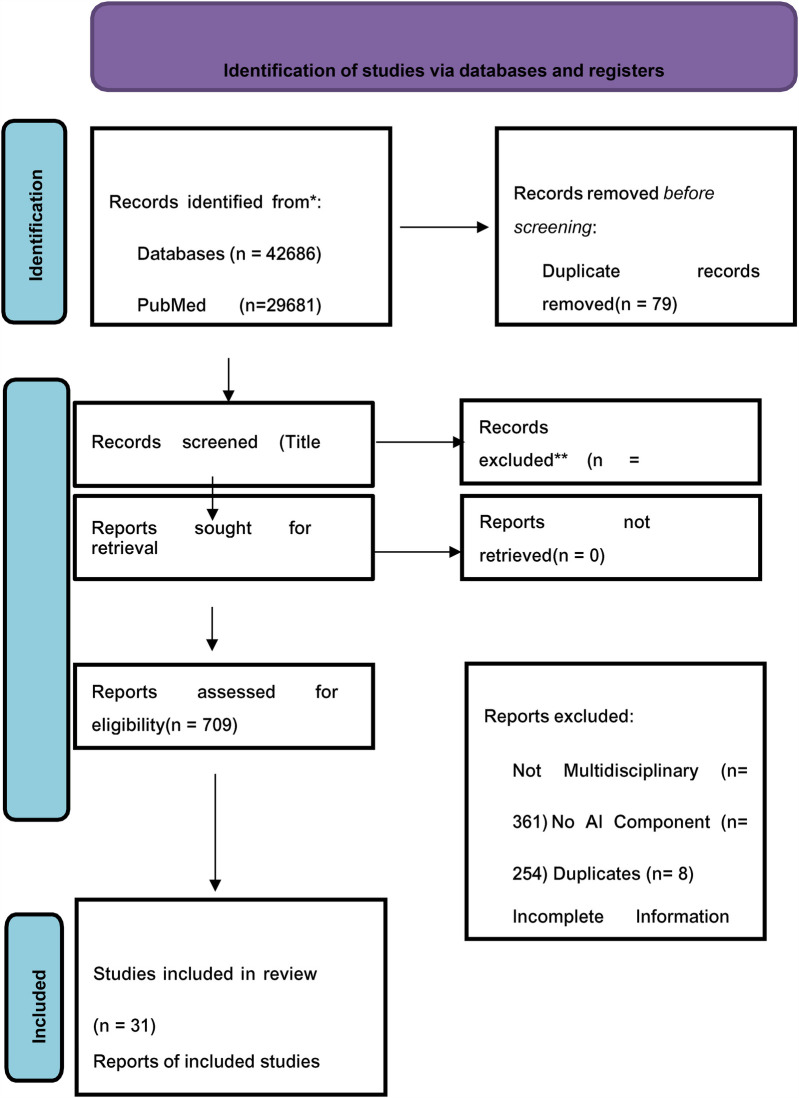
PRISMA flowchart showing the study selection process.

The preliminary search identified a total of 42,686 articles, from which 79 duplicates were removed. Following a rigorous screening of titles and abstracts, 41,792 articles were excluded based on predetermined criteria, resulting in 709 articles that were selected for a full-text review. After a detailed evaluation, 678 articles were excluded due to not meeting the eligibility standards, some for multiple reasons. The specific reasons for exclusion are detailed in the PRISMA flowchart provided below. Ultimately, only 31 articles were deemed appropriate for inclusion in this review paper. The study selection process is depicted in Figure 1.

Although the strategy of research, eligibility criteria, study selection process, and data extraction methods are described, the inclusion and exclusion criteria utilized during the review process should be elaborated further. Providing additional details on these criteria would enhance the transparency of the study, allowing readers to better assess the robustness of the methodology.

The inclusion criteria for the study were:

• Original studies.
• Studies published between 2013 and August 2023, reflecting the most recent research.
• Focus on multidisciplinary AI.
• Studies that contribute to the understanding of how multidisciplinary collaboration shapes AI development.
• Published in English.

The exclusion criteria were:

• Studies not directly related to the multidisciplinary approach in AI development, including those focusing solely on the technical aspects of AI without interdisciplinary collaboration.
• Non-English studies.
• Non-peer-reviewed sources, such as reviews, editorials, conference papers, and comments.
• Abstracts or summaries that lacked sufficient detail to evaluate their relevance to the review objectives.
• Studies concentrating exclusively on a single aspect of AI (e.g., natural language processing, computer vision) without discussing its multidisciplinary integration.

### 3.1 Characteristics of included articles

Data extraction results are shown in Table 1.

### 3.2 Included studies

This systematic review comprises 31 studies: 13 qualitative, 13 quantitative, and 5 utilizing mixed methodologies. The review encompasses a variety of disciplines, encompassing mathematics, medicine, psychology, physics, agriculture, and economics.

#### 3.2.1 Heterogeneity assessment

The potential heterogeneity among the included studies was evaluated to understand variations in study results and their implications for the overall conclusions of this review. Visual inspection of forest plots, where applicable, was conducted to identify patterns of variability in effect sizes across studies. Statistical measures such as the chi-square test (Cochran's *Q* test) and the I^2^ statistic were also employed to quantify the extent of heterogeneity.

The I^2^ statistic was interpreted as follows:

0%–25%: low heterogeneity,

25%–50%: moderate heterogeneity,

50%–75%: substantial heterogeneity, and

75%: considerable heterogeneity.

When significant heterogeneity (I^2^ > 50%) was detected, subgroup analyses or meta-regressions were considered to explore potential sources of variability, such as differences in study populations, methodologies, or intervention types. The findings related to heterogeneity are presented are providing insights into the consistency of evidence across the included studies. The assessment of heterogeneity not only informed the decision on whether to pool study results but also enhanced the interpretability of the synthesized evidence, ensuring a more nuanced understanding of the multidisciplinary impact of artificial intelligence.

### 3.3 Thematic analysis

This thematic analysis was undertaken to explore the multidisciplinary approach to artificial intelligence through an in-depth examination of six distinct disciplines. The intersection of AI with mathematics, medicine, psychology, physics, agriculture, and economics has yielded a panorama of innovation, redefining conventional boundaries and catalyzing unprecedented advancements.

#### 3.3.1 AI in medicine and healthcare

The application of AI within the medical and healthcare sectors is ushering in a new era of precision diagnostics, personalized treatments, and improved patient outcomes. Delving into this theme, we examine how AI algorithms analyze medical imaging data, aid in disease diagnosis, and assist medical professionals in treatment planning. A qualitative study by Ahuja et al. (8) stated that AI systems designed for ophthalmology offer benefits by reducing the time needed for image data analysis. They also empower ophthalmologists to enhance their comprehension of how diseases advance and aid in the early detection, classification, and prediction of various stages of these diseases. Butow and Hoque (9), conveyed that efforts have been made to apply artificial intelligence in healthcare for training communication skills. This has been done to offer assessment and constructive feedback, along with utilizing avatars as a part of this process. Ferreira et al. (10) demonstrated the possible advantages of introducing aspiring scientists, engineers, and clinicians to cross-cultural design thinking during their educational journeys, highlighting its potential contributions to the integration of AI in the medical field.

A qualitative study by Gruson et al. (11) how the integration of AI and laboratory medicine acts as a catalyst for personalized care, enhancing precision and the demand for value-driven services within the realm of cardiovascular medicine. Hu et al. (12) reported how AI displayed significant promise in extending the boundaries of microscopy and elevating the quality of acquired data by improving resolution, signal strength, and information richness. A quantitative study by Luo et al. (13) investigated whether a high-capacity, real-time automated system for detecting polyps could enhance the polyp detection rate (PDR) within a genuine clinical setting. The researchers found that the AI system notably raised the PDR (34.0% vs. 38.7%, *p* < 0.001). Moreover, the use of AI-assisted colonoscopy led to an increase in the detection of polyps smaller than 6 mm. Makino et al. (36) deduced that the new predictive AI model could identify the advancement of diabetic kidney diseases (DKD). This advancement could potentially lead to more precise and efficient interventions aimed at reducing the need for hemodialysis. A study by Nishida and Kudo (14) reported that certain AI models have been documented to surpass the performance of human experts, suggesting their capacity to aid clinical practice due to their rapid and efficient results. Reddy et al. (15) indicated that AI's most substantial impact would be observed in four primary domains: patient management, aiding clinical decisions, patient surveillance, and healthcare interventions because of its integration.

A quantitative study by Rondonotti et al. (16) reported that AI-supported optical diagnosis meets the necessary PIVI criteria, although it doesn't eliminate the necessity for endoscopists’ substantial confidence and proficiency. The AI system appears to hold value, particularly for individuals without extensive expertise. Sezgin (17) concluded that AI has the potential to bring about a fundamental change in healthcare by supplementing and augmenting the capabilities of healthcare professionals.

This could result in heightened service quality, enhanced patient outcomes, and a more streamlined healthcare system. Shandhi and Dunn (18) reported that progress in AI is facilitating the customization of healthcare. This includes tasks like exploring the genetic or molecular basis of diseases, comprehending individual responses to medications, and integrating various personal data sources such as physiological, behavioural, laboratory, and clinical information to reveal novel insights into the underlying mechanisms of diseases. Sonoda et al. (19) examined the level of agreement in the running pattern of choroidal vessels between an artificial intelligence (AI) system and experienced clinicians. The authors concluded that the AI algorithm was capable of automatically determining the presence or absence of a symmetrical blood vessel running pattern in the choroid, even in situations with unclear criteria. The accuracy and consistency of this classification were comparable to those achieved by skilled human experts. Williams et al. (20) reported that employing advanced AI in combination with physiological genomic measurements in tissues relevant to diseases can offer potent strategy for achieving precision medicine in common health conditions.

In summary, Artificial Intelligence aims to imitate human cognition processes, in derstanding an in the presentation of complex clinical and medical data. As a result, when researchers, scientists and healthcare teams enter data into PCs, algorithms can analyze it, interpret it, and AI immediately proposes solutions to complex problems. AI can be used to improve speed and the accuracy of disease diagnosis and screening. This can create positive effects on the progronis of serious diseases; strengthen clinical research and development of new pharmacological and non-pharmacological therapies.

AI can be a resource for better management of healthcare systems. Finally, with this system, it is possible to significantly reduce the costs both for patients and for the healthcare system (Digital Health 2030).

#### 3.3.2 Cognitive and psychological aspects of AI

AI's integration extends beyond algorithms; it touches the realm of human cognition and psychology. This theme explores how AI's presence impacts human-machine interactions, leading to the rise of virtual assistants, recommendation systems, and sentiment analysis. A study by Bird et al. (21) shares the outcomes of an internet-based randomized controlled trial involving Manage Your Life Online (MYLO), a system utilizing artificial intelligence to interact with users about various issues. The authors discovered that MYLO received notably higher ratings for usefulness compared to ELIZA. However, there was no significant overall impact of the intervention on resolving problems. A study by Choi et al. (22) assessed the efficiency of a mobile application called Circadian Rhythm for Mood (CRM). The app was created to proactively prevent mood fluctuations using a machine learning algorithm that analyzes passive digital behavioural data related to circadian rhythms. This data is collected through a wearable activity tracker. The authors noted that the CRM group experienced favourable alterations in health-related behaviours due to the alerts, and there was an increase in adherence rates to wearable devices. A study by Fulmer et al. (23) evaluated whether employing an integrated psychological AI named Tess could effectively alleviate self-identified symptoms of depression and anxiety in college students. The authors concluded that AI, in this capacity, could offer cost-effective and easily accessible therapeutic assistance. While not intended to replace the role of a trained therapist, the use of integrated psychological AI emerged as a practical approach to providing support. Green et al. (24) collected initial information about the Healthy Moms intervention for perinatal depression, intending to understand how to develop and evaluate a stronger program. The authors reported that the majority of participants who used Zuri, the intervention service, conveyed a favourable outlook towards it and demonstrated confidence in its effectiveness.

A mixed study by Inkster et al. (25) provided an initial assessment of real-world data concerning the efficacy and user engagement of a text-based conversational mobile app, Wysa. This app incorporated AI and empathy to support mental well-being and was tested on individuals who self-reported symptoms of depression. The authors’ conclusion highlighted the potential seen in the real-world data evaluation results of the Wysa app for users with self-reported depression symptoms. Liu et al. (26) concluded that the self-help depression intervention provided through a chatbot surpassed the minimal bibliotherapy level in terms of effectively reducing depression and anxiety symptoms, as well as fostering a stronger therapeutic alliance with the participants. A study by Piette et al. (27) aimed to establish whether a personalized patient treatment program, utilizing reinforcement learning from the field of artificial intelligence (AI) and interactive voice response (IVR) calls, is as effective as traditional telephone-based CBT-CP and economizes therapist time. The researchers concluded that a higher percentage of patients undergoing AI-CBT-CP exhibited clinically significant improvements at the 6-month mark, demonstrated by RMDQ scores (37% vs. 19%; *P* = .01) and pain intensity scores (29% vs. 17%; *P* = .03). Sadeh-Sharvit et al. (28) examined whether an AI platform designed for behavioral health could viably improve clinical results for individuals undergoing outpatient therapy, while also assessing its acceptability. The authors concluded that enhancing mental health services in community clinics with a specialized AI platform for behavioural treatment yielded greater efficacy in reducing significant symptoms compared to conventional therapy.

Psychological interventions with AI are therefore effective in many contexts. The virtual psychotherapist is available from Monday to Sunday, 24 hours a day. The therapist can be in any place near or far from the patient (home, hospital, room, etc.).

These procedures can complement standard clinical interventions with new strategies and procedures. Clinical treatment can be useful in situations of mild discomfort and in situations of general malaise.

Psychological support treatment could provide primary care services to people who have difficulty accessing primary healthcare services.

We could conclude saying that the supposed psychological treatment with AI is a resource to be added to the traditional psychological support path. In fact a further advantage is the better relation between cost and benefit; numerous studies report a reduction of health care costs.

#### 3.3.3 AI in agriculture and food security

In the agricultural domain, AI's transformative potential is harnessed to address pressing global challenges such as food security and sustainable farming practices. Ben and Hanan (29) showcased the primary uses of AI and ML algorithms within various segments of the agricultural supply chain. They highlighted the undeniable upward trend in adopting these algorithms to enhance the food industry. A study by Montazeri et al. (30) utilized artificial intelligence to present an innovative structure for simulating the trajectory, movement, and assessment of chromium starting from its point of release into a river until its absorption by crops. The AI models tested encompassed linear regression (LR), a neural network (NN) classifier, and an NN regressor. The authors concluded that among these, the NN regressor exhibited the highest precision in estimating chromium levels within tomato leaves, with LR following as the next accurate option.

#### 3.3.4 AI in mathematics

AI's journey begins with the fundamental algorithms, statistical methods, and mathematical models that power its predictive capabilities. This discipline provides the bedrock upon which machine learning and deep learning frameworks are constructed. A study by Ahmad and Afzal (31), discovered that a mathematical representation of individuals suspected of having COVID-19. The representation took the shape of a multi-criteria decision-making (MCDM) model, accompanied by an innovative artificial intelligence method. It successfully developed and put into action this approach using newly formulated measures of plithogenic distance and similarity in a fuzzy setting. Davies et al. (32) presented a collaboration model between the realms of mathematics and artificial intelligence, designed to assist mathematicians in uncovering novel conjectures and theorems. A study by Elleuch et al. (33) suggested that merging two approaches—Artificial Intelligence (AI) using Artificial Neural Networks (ANN), and Operations Research (OR) using a Fuzzy Interval Mathematical model—proved to be a potent strategy for evaluating a suitable list of patients for a group of hospitals, particularly when faced with limited initial information.

#### 3.3.5 AI and its applications in physics

The intersection of AI and physics has yielded innovative approaches to understanding complex physical phenomena and harnessing the power of data-driven insights. Diaz et al. (34) assessed existing attitudes, methods, and educational requirements regarding artificial intelligence within the domain of medical physics. The authors deduced that AI was viewed as a valuable tool for aiding Medical Physics Experts (MPEs) in their everyday responsibilities. Respondents expressed substantial enthusiasm for enhancing their current AI-related competencies, underscoring the necessity for specialized training tailored to MPEs.

#### 3.3.6 AI in economics

The fusion of AI and economics shapes a landscape characterized by automation, innovation, and evolving labour dynamics. A qualitative study by Sohrabpour et al. (35) conveyed that a new AI- driven causal structure designed for modelling and predicting export sales could anticipate market patterns and enhance the allocation of resources.

## 4 Discussion

In the swiftly evolving realm of artificial intelligence (AI), a striking trend has emerged, emphasizing the crucial role of collaboration across diverse disciplines. The multidisciplinary approach to AI represents a departure from isolated technological development and embraces the integration of various fields, including computer science, medicine, psychology, agriculture, mathematics, physics, and economics. This study aims to contribute to this understanding by delving into a systematic review that meticulously examines the multidisciplinary approach to AI development by synthesizing findings from studies spanning various domains.

### 4.1 Scope and prospects of multidisciplinary AI

The scope of AI's influence extends beyond the specific applications discussed in this review, and the prospects for its future integration across disciplines are vast. AI's potential to revolutionize various sectors is only beginning to be understood. For instance, in medicine, AI's role could evolve from assisting with diagnostics to providing comprehensive patient management systems that integrate real-time data from multiple sources, enabling more personalized and efficient care. In agriculture, AI could play a critical role in addressing global food security by optimizing supply chains and predicting crop yields with greater accuracy. Moreover, as AI technologies continue to develop, we may see their application in new and emerging fields, such as environmental science, where AI could help monitor and mitigate the impacts of climate change.

Looking forward, the multidisciplinary approach to AI holds promise for addressing some of the most pressing global challenges. For example, the intersection of AI with public health could pave the way for more effective management of future pandemics through predictive modeling and rapid response systems. In education, AI could provide tailored learning experiences that adapt to individual student needs, potentially reducing educational disparities.

### 4.2 Practical implications of multidisciplinary AI developments

The integration of artificial intelligence within the medical and healthcare sectors represents a significant stride toward precision diagnostics, personalized treatments, and improved patient outcomes. The reviewed studies collectively emphasize the transformative potential of AI in diverse medical domains. For instance, Ahuja et al. (8) highlighted how AI systems specializing in ophthalmology expedite image analysis, aiding in early disease detection and classification. This aligns with the findings of Nishida and Kudo (14), who underscored the potential for certain AI models to surpass human experts’ performance, indicating their role as a rapid and efficient clinical support tool.

The practical implications of the integration of AI across disciplines are significant. In real-world scenarios, AI-driven innovations can transform practices across industries. In healthcare, the deployment of AI tools could lead to earlier and more accurate diagnoses, thereby improving patient outcomes and reducing healthcare costs. For instance, AI systems could be integrated into hospital workflows to assist clinicians in decision-making processes, ensuring that patient care is both timely and effective.

In the agricultural sector, AI has the potential to enhance food production systems by improving the efficiency of farming practices. This could involve the use of AI-driven machinery that adapts to environmental conditions in real-time, thus maximizing yield and minimizing waste. Furthermore, AI's role in environmental management, such as monitoring water quality or predicting the spread of pollutants, highlights its potential to contribute to sustainable development goals.

However, the implementation of AI technologies is not without challenges. There are significant ethical and regulatory considerations that must be addressed, particularly in sectors like healthcare, where the stakes are high. Ensuring that AI systems are transparent, accountable, and free from bias is crucial to gaining public trust and ensuring the equitable distribution of AI's benefits. Policymakers will need to establish frameworks that promote innovation while safeguarding against potential risks associated with AI.

Moreover, the application of AI in medical imaging, as demonstrated by Hu et al. (12), offers enhanced microscopy capabilities, leading to higher data quality and resolution. Studies such as Luo et al. (13) showcase the utility of AI in real-time automated systems, resulting in improved detection rates for conditions like polyps. Such findings indicate AI's ability to augment clinical decision-making and patient management.

The integration of AI in the medical field is not limited to diagnostics alone. Ferreira et al. (10) emphasize the benefits of cross-cultural design thinking, fostering collaboration between scientists, engineers, and clinicians. This highlights the role of AI in promoting interdisciplinary collaboration and innovative approaches. Beyond medical applications, the influence of AI extends into cognitive and psychological realms. Several studies underscore the potential of AI-driven interventions for mental health support. Fulmer et al. (23) showcased the promise of integrated psychological AI platforms in alleviating symptoms of depression and anxiety, providing easily accessible therapeutic assistance. These findings suggest that while not a substitute for trained therapists, integrated psychological AI can play a pivotal role in enhancing mental health care accessibility. Similarly, Choi et al. (22) highlighted the efficacy of AI-driven applications in managing mood fluctuations by analyzing circadian rhythms. The positive effects observed in health-related behaviours and adherence rates reflect the potential of AI to provide proactive interventions for individuals’ well-being. An area notably absent in the current scope of this systematic review is the integration of artificial intelligence in educational testing and assessment, closely related to psychological measurement and testing. Educational testing is a critical field with implications across various domains, including cognitive psychology, pedagogy, and workforce development. AI has demonstrated significant potential in enhancing the accuracy, efficiency, and adaptability of educational assessments. For example, AI-driven adaptive testing systems can tailor the difficulty of questions to a test-taker's ability in real-time, improving both engagement and diagnostic accuracy. Furthermore, AI has enabled the automation of scoring for open-ended responses and essay-type questions, a task traditionally requiring extensive human input. These applications not only streamline testing processes but also provide valuable insights into learners’ cognitive processes and areas needing intervention. By integrating educational testing into the multidisciplinary AI framework, this review could highlight its impact on fostering equitable access to quality education, personalizing learning experiences, and addressing global educational challenges. Future iterations of this review should aim to explore this domain, given its profound implications for advancing both individual learning outcomes and broader societal goals.

In the agricultural sector, AI's integration emerges as a key strategy for addressing challenges like food security and sustainable farming practices. The work of Ben and Hanan (29) emphasizes the growing trend of utilizing AI and machine learning across various segments of the agricultural supply chain. This trend has the potential to enhance the entire food industry, showcasing AI's role in supporting global sustainability goals. Furthermore, the application of AI in simulating the movement of pollutants through crops, as seen in Montazeri et al. (30), highlights how AI models can aid in environmental management and risk assessment, contributing to informed decision-making.

The intersection of AI with fundamental disciplines like mathematics, physics, and economics brings forth novel approaches and insights. Studies such as Ahmad and Afzal (31) demonstrate the integration of mathematical models and AI techniques to address real-world challenges like COVID-19 diagnosis. This synergy showcases the potential of AI to enhance problem-solving in complex domains. Davies et al. (32) present a collaborative model between mathematics and AI, enabling the discovery of conjectures and theorems. Such partnerships between traditional disciplines and AI pave the way for innovative discoveries and solutions.

The multidisciplinary nature of AI's applications is evident across the reviewed studies. The collaborative efforts between AI and various fields, including medicine, psychology, agriculture, and mathematics, underscore the potential of interdisciplinary approaches to drive innovation and transformative change. Looking ahead, the findings of Sezgin (17) and Shandhi and Dunn (18) suggest that AI's continued integration could revolutionize healthcare by augmenting healthcare professionals’ capabilities, improving service quality, and streamlining healthcare systems. In agriculture, AI-driven solutions hold promise for addressing food security challenges and sustainable farming practices. Furthermore, the intersection of AI with mathematics, physics, and economics promises to reshape problem-solving methodologies and insights generation.

### 4.3 Future directions and interdisciplinary collaboration

The future of AI lies in continued interdisciplinary collaboration. The convergence of expertise from different fields is essential to unlocking AI's full potential. For instance, collaborations between computer scientists and medical professionals can lead to the development of AI systems that are not only technically advanced but also clinically relevant. Similarly, partnerships between AI researchers and experts in agriculture can lead to innovations that address the specific challenges faced by farmers.

Educational initiatives will play a crucial role in fostering these collaborations. Training programs that encourage interdisciplinary learning and research can help bridge the gap between disciplines, ensuring that professionals are equipped with the knowledge and skills needed to harness AI's potential. This is particularly important as AI technologies become increasingly complex and integrated into various aspects of society.

It's important to acknowledge certain limitations of the reviewed studies. Variability in study methodologies, sample sizes, and contexts may impact the generalizability of findings. Additionally, the rapidly evolving nature of AI technology and its multidisciplinary applications could render some studies outdated or incomplete in capturing the full scope of AI's impact.

The reviewed studies collectively illuminate the transformative potential of a multidisciplinary approach to AI. As AI permeates diverse fields, it catalyzes innovation, enhances problem-solving, and reshapes conventional practices. The integration of AI across medicine, psychology, agriculture, mathematics, physics, and economics underscores the breadth of its influence. Collaborations at the intersection of AI and these disciplines hold the promise of pushing the boundaries of knowledge and driving positive change on a global scale. As AI's journey continues, its multidisciplinary approach remains a beacon of advancement and discovery.

### 4.4 Quality of evidence

The quality of evidence across the included studies was systematically evaluated using the GRADE framework. This method enabled a thorough assessment of the robustness and reliability of findings, taking into account key factors such as study design, consistency of results, precision, directness, and potential biases. The GRADE approach categorized evidence levels as high, moderate, low, or very low, reflecting the confidence in the applicability and accuracy of the reported outcomes.

While several studies demonstrated strong methodological designs and consistent findings, others exhibited limitations, such as small sample sizes or potential risks of bias, which impacted the overall strength of evidence. For example, studies that relied heavily on self-reported measures or lacked proper randomization were assigned lower evidence grades. Conversely, well-conducted randomized controlled trials with robust methodologies contributed high-quality evidence to the review.

This variability in evidence quality underscores the need for caution in interpreting and generalizing results. Future studies should aim to adopt rigorous designs and standardized methodologies to strengthen the evidence base, ensuring that findings are both reliable and applicable across diverse contexts.

## 5 Limitations of the study

While this review has highlighted the transformative potential of AI, it is important to acknowledge the limitations of the study. The variability in study methodologies, sample sizes, and contexts across the reviewed literature may impact the generalizability of the findings. Additionally, the rapid pace of AI development means that some studies may become outdated quickly, as new technologies and applications emerge.

Moreover, the focus of this review on certain disciplines may have inadvertently overlooked other areas where AI could have a significant impact. Future research should aim to explore these underrepresented fields and provide a more comprehensive understanding of the multidisciplinary applications of AI. Additionally, more attention should be given to the ethical, legal, and social implications of AI, particularly as it becomes more integrated into daily life.

This systematic review, while comprehensive in scope, has certain limitations that must be acknowledged. First, the variability in study designs, methodologies, and sample sizes across the included studies presents challenges in comparing and synthesizing results. This heterogeneity may affect the generalizability of the findings and limit the ability to draw definitive conclusions. Additionally, while this review adhered to PRISMA guidelines, the lack of protocol registration at the onset of the study is a notable omission that could influence transparency and reduce the ability to replicate the methodology.

The review also lacks a formal assessment of publication bias, which could affect the interpretation of the results. Furthermore, the exclusion of non-English studies may have resulted in a language bias, potentially omitting significant contributions from non-English literature. Finally, certain fields, such as educational testing and assessment, are underrepresented in the reviewed studies, despite their critical intersection with artificial intelligence and psychological measurement.

## 6 Conclusion

This systematic review provided a comprehensive insight into the transformative power of the multidisciplinary approach to artificial intelligence. The convergence of AI with diverse disciplines such as medicine, psychology, agriculture, mathematics, physics, art and economics unveils a new era of innovation and collaboration. In addition, the decision-making process and the time required, to implement significant changes through new technologies, are important for different contexts. The development of digital devices, robots, and direct online access to a wide range of information sources aim to improve the speed and quality of outcomes.

However, a major challenge for professionals is to maintain an overview of the digital possibilities and tools in order to use them efficiently in every field of work, subsequent to appropriate training, on the use and development of AI.

The studies showcased within this review collectively underscored the multifaceted impact of AI on various disciplines. From revolutionizing medical diagnostics and treatment planning to enhancing mental health interventions, and from addressing global challenges in agriculture to reshaping problem- solving in mathematics and physics, AI's integration is reshaping conventional practices and expanding the horizons of knowledge. As AI continues its journey, the multidisciplinary approach remains a testament to its potential to drive positive change, revolutionize industries, and offer unprecedented insights into complex challenges. In an increasingly interconnected world, the fusion of AI with diverse fields holds the promise of ushering in an era of interdisciplinary collaboration that has the potential to redefine the limits of human achievement and transform the way we approach critical issues facing humanity. The scientific organisations should “advance scientific understanding of the world, and to enable the application of this knowledge for the benefit and betterment of humankind” (IIIM, 2015).

## Data Availability

The original contributions presented in the study are included in the article/Supplementary Material, further inquiries can be directed to the corresponding author/s.

## References

[1] Berente N Gu B Recker J Santhanam R. Managing artificial intelligence. MIS Q. (2021) 45(3):1433–50. 10.25300/MISQ/2021/16274

[2] Jordan MI. Artificial intelligence—the revolution hasn’t happened yet. Harv Data Sci Rev. (2019) 1(1):1–9. 10.1162/99608f92.f06c6e61

[3] Zhang C Lu Y. Study on artificial intelligence: the state of the art and future prospects. J Ind Inf Integr. (2021) 23:100224. 10.1016/j.jii.2021.100224

[4] Esteva A Chou K Yeung S Naik N Madani A Mottaghi A Deep learning-enabled medical computer vision. NPJ Digit Med. (2021) 4(1):5. 10.1038/s41746-020-00376-2 33420381 PMC7794558

[5] Zeng X Wang F Luo Y Kang SG Tang J Lightstone FC Deep Generative Molecular Design Reshapes Drug Discovery. Cambridge, MA: Cell Press (2022). 10.1016/j.xcrm.2022.100794 PMC9797947 36306797

[6] Grewal D Hulland J Kopalle PK Karahanna E. The future of technology and marketing: a multidisciplinary perspective. J Acad Mark Sci. (2020) 48:1–8. 10.1007/s11747-019-00711-4

[7] Page MJ McKenzie JE Bossuyt PM Boutron I Hoffmann TC Mulrow CD The PRISMA 2020 statement: an updated guideline for reporting systematic reviews. BMJ. (2021) 372:n71. 10.1136/bmj.n71 33782057 PMC8005924

[8] Ahuja AS Wagner IV Dorairaj S Checo L Hulzen RT. Artificial intelligence in ophthalmology: a multidisciplinary approach. Integr Med Res. (2022) 11(4):100888. 10.1016/j.imr.2022.100888 36212633 PMC9539781

[9] Butow P Hoque E. Using artificial intelligence to analyse and teach communication in healthcare. Breast. (2020) 50:49–55. 10.1016/j.breast.2020.01.008 32007704 PMC7375542

[10] Ferreira MF Savoy JN Markey MK. Teaching cross-cultural design thinking for 470 healthcare. Breast. (2020) 50:1–10. 10.1016/j.breast.2019.12.015 31958660 PMC7375602

[11] Gruson D Bernardini S Dabla PK Gouget B Stankovic S. Collaborative AI and laboratory medicine integration in precision cardiovascular medicine. Clin Chim Acta. (2020) 509:67–71. 10.1016/j.cca.2020.06.001 32505771

[12] Hu Y Luo Y Tang G Huang Y Kang J Wang D. Artificial intelligence and its applications in digital hematopathology. Blood Sci. (2022) 4(3):136–42. 10.1097/BS9.0000000000000130 36518598 PMC9742095

[13] Luo Y Zhang Y Liu M Lai Y Liu P Wang Z Artificial intelligence-assisted colonoscopy for detection of colon polyps: a prospective, randomized cohort study. J Gastrointest Surg. (2021) 25(8):2011–8. 10.1007/s11605-020-04802-4 32968933 PMC8321985

[14] Nishida N Kudo M. Artificial intelligence models for the diagnosis and management of liver diseases. Ultrasonography. (2023) 42(1):10–9. 10.14366/usg.22110 36443931 PMC9816706

[15] Reddy S Fox J Purohit MP. Artificial intelligence-enabled healthcare delivery. J R Soc Med. (2019) 112(1):22–8. 10.1177/0141076818815510 30507284 PMC6348559

[16] Rondonotti E Hassan C Tamanini G Antonelli G Andrisani G Leonetti G Artificial intelligence-assisted optical diagnosis for the resect-and-discard strategy in clinical practice: the artificial intelligence BLI characterization (ABC) study. Endoscopy. (2023) 55(1):14–22. 10.1055/a-1852-0330 35562098

[17] Sezgin E. Artificial intelligence in healthcare: complementing, not replacing, doctors and healthcare 554 providers. Digit Health. (2023) 9:20552076231186520. 10.1177/20552076231186520 37426593 PMC10328041

[18] Shandhi MMH Dunn JP. AI in medicine: where are we now and where are we going? Cell Rep Med. (2022) 3(12):100861. 10.1016/j.xcrm.2022.100861 36543109 PMC9798019

[19] Sonoda S Shiihara H Terasaki H Kakiuchi N Funatsu R Tomita M Artificial intelligence for classifying uncertain images by humans in determining choroidal vascular running pattern and comparisons with automated classification between artificial intelligence. PLoS One. (2021) 16(5):e0251553. 10.1371/journal.pone.0251553 33989334 PMC8121314

[20] Williams AM Liu Y Regner KR Jotterand F Liu P Liang M. Artificial intelligence, physiological genomics, and precision medicine. Physiol Genomics. (2018) 50(4):237–43. 10.1152/physiolgenomics.00119.2017 29373082 PMC5966805

[21] Bird T Mansell W Wright J Gaffney H Tai S. Manage your life online: a web-based randomized controlled trial evaluating the effectiveness of a problem-solving intervention in a student sample. Behav Cogn Psychother. (2018) 46(5):570–82. 10.1017/s1352465817000820 29366432

[22] Cho C-H Lee T Lee J-B Seo JY Jee H-J Son S Effectiveness of a smartphone app with a wearable activity tracker in preventing the recurrence of mood disorders: prospective case-control study. JMIR Ment Health. (2020) 7(8):e21283. 10.2196/21283 32755884 PMC7439135

[23] Fulmer R Joerin A Gentile B Lakerink L Rauws M. Using psychological artificial intelligence (tess) to relieve symptoms of depression and anxiety: randomized controlled trial. JMIR Ment Health. (2018) 5(4):e64. 10.2196/mental.9782 30545815 PMC6315222

[24] Green EP Lai Y Pearson N Rajasekharan S Rauws M Joerin A Expanding access to perinatal depression treatment in Kenya through automated psychological support: development and usability study. JMIR Form Res. (2020) 4(10):e17895. 10.2196/17895 33016883 PMC7573703

[25] Inkster B Sarda S Subramanian V. An empathy-driven, conversational artificial intelligence agent (wysa) for digital mental well-being: real-world data evaluation mixed-methods study. JMIR Mhealth Uhealth. (2018) 6(11):e12106. 10.2196/12106 30470676 PMC6286427

[26] Liu H Peng H Song X Xu C Zhang M. Using AI chatbots to provide self-help depression interventions for university students: a randomized trial of effectiveness. Internet Interv. (2022) 27:100495. 10.1016/j.invent.2022.100495 35059305 PMC8760455

[27] Piette JD Newman S Krein SL Marinec N Chen J Williams DA Patient-Centered pain care using artificial intelligence and Mobile health tools. JAMA Intern Med. (2022) 182(9):975. 10.1001/jamainternmed.2022.3178 35939288 PMC9361183

[28] Sadeh-Sharvit S Camp TD Horton SE Hefner JD Berry JM Grossman E Effects of an artificial intelligence platform for behavioral interventions on depression and anxiety symptoms: randomized clinical trial. J Med Internet Res. (2023) 25:e46781. 10.2196/46781 37428547 PMC10366966

[29] Ben Ayed R Hanana M. Artificial intelligence to improve the food and agriculture sector. J Food Qual. (2021) 2021:1–7. 10.1155/2021/5584754

[30] Montazeri A Chahkandi B Gheibi M Eftekhari M Wacławek S Behzadian K A novel AI-based approach for modelling the fate, transportation and prediction of chromium in rivers and agricultural crops: a case study in Iran. Ecotoxicol Environ Saf. (2023) 263:115269–115269. 10.1016/j.ecoenv.2023.115269 37478568

[31] Ahmad MR Afzal U. Mathematical modeling and AI-based decision making for COVID-19 suspects backed by novel distance and similarity measures on plithogenic hypersoft sets. Artif Intell Med. (2022) 132:102390. 10.1016/j.artmed.2022.102390 36207091 PMC9436789

[32] Davies A Veličković P Buesing L Blackwell S Zheng D Tomašev N Advancing mathematics by guiding human intuition with AI. Nature. (2021) 600(7887):70–4. 10.1038/s41586-021-04086-x 34853458 PMC8636249

[33] Elleuch MA Hassena AB Abdelhedi M Pinto FS. Real-time prediction of COVID-19 patients’ health situations using artificial neural networks and fuzzy interval mathematical modeling. Appl Soft Comput. (2021) 110:107643. 10.1016/j.asoc.2021.107643 34188610 PMC8225317

[34] Diaz O Guidi G Ivashchenko O Colgan N Zanca F. Artificial intelligence in the medical physics community: an international survey. Phys Med. (2021) 81:141–6. 10.1016/j.ejmp.2020.11.037 33453506

[35] Sohrabpour V Oghazi P Toorajipour R Nazarpour A. Export sales forecasting using artificial intelligence. Technol Forecast Soc Change. (2020) 163:120480. 10.1016/j.techfore.2020.120480

[36] Makino M Yoshimoto R Ono M Itoko T Katsuki T Koseki A Artificial intelligence predicts the progression of diabetic kidney disease using big data machine learning. Sci Rep. (2019) 9:11862. 10.1038/s41598-019-48263-5 31413285 PMC6694113

